# Exploring Interaction between Genetically Predicted Body Mass Index and Serum 25-Hydroxyvitamin D Levels on the Odds for Psoriasis in UK Biobank and the HUNT Study: A Factorial Mendelian Randomization Study

**DOI:** 10.1016/j.xjidi.2024.100336

**Published:** 2024-12-04

**Authors:** Marita Jenssen, Nikhil Arora, Mari Løset, Bjørn Olav Åsvold, Laurent Thomas, Ole-Jørgen Bekkevold Vassmyr, Xiao-Mei Mai, Yi-Qian Sun, Anne-Sofie Furberg, Rolf Jorde, Tom Wilsgaard, Kjersti Danielsen, Ben Michael Brumpton

**Affiliations:** 1 Department of Dermatology, University Hospital of North Norway, Tromsø, Norway; 2 Department of Community Medicine, UiT The Arctic University of Norway, Tromsø, Norway; 3 HUNT Center for Molecular and Clinical Epidemiology, Department of Public Health and Nursing, Norwegian University of Science and Technology (NTNU), Trondheim, Norway; 4 Population Health Sciences, Bristol Medical School, University of Bristol, Bristol, United Kingdom; 5 Department of Dermatology, Clinic of Orthopedy, Rheumatology and Dermatology, St. Olavs Hospital, Trondheim University Hospital, Trondheim, Norway; 6 HUNT Research Centre, Department of Public Health and Nursing, Norwegian University of Science and Technology, Levanger, Norway; 7 Department of Endocrinology, Clinic of Medicine, St. Olavs Hospital, Trondheim, Norway; 8 Department of Public Health and Nursing, Norwegian University of Science and Technology (NTNU), Trondheim, Norway; 9 Department of Clinical and Molecular Medicine, Norwegian University of Science and Technology (NTNU), Trondheim, Norway; 10 Clinic of Laboratory Medicine, Department of Pathology, St. Olavs Hospital, Trondheim, Norway; 11 Center for Oral Health Services and Research Mid-Norway (TkMidt), Trondheim, Norway; 12 Department of Microbiology and Infection Control, University Hospital of North Norway, Tromsø, Norway; 13 Faculty of Health Sciences and Social Care, Molde University College, Molde, Norway; 14 Department of Clinical Medicine, UiT The Arctic University of Norway, Tromsø, Norway

**Keywords:** BMI, Interaction, Mendelian randomization, Psoriasis, Vitamin D

## Abstract

Mendelian randomization (MR) studies show that higher body mass index (BMI) and lower 25-hydroxyvitamin D (25[OH]D) increase psoriasis risk. The combined effect of these factors has not been explored using factorial MR. Using cross-sectional data from UK Biobank (n = 398,404) and The Trøndelag Health Study (n = 86,648), we calculated polygenic risk scores for BMI and 25(OH)D to estimate ORs for psoriasis using 2 × 2 and continuous factorial MR. We quantified additive interaction by estimating relative excess risk due to interaction. We also performed traditional observational analyses in UK Biobank. There were 12,207 (3.1%) participants with psoriasis in UK Biobank and 7794 (9.0%) in The Trøndelag Health Study. In 2 × 2 factorial MR, we found no evidence of relative excess risk for psoriasis due to interaction between genetically predicted higher BMI and lower 25(OH)D, neither in UK Biobank (relative excess risk due to interaction = −0.01, 95% confidence interval = −0.08 to 0.07) nor in The Trøndelag Health Study (relative excess risk due to interaction = −0.04, 95% confidence interval = −0.14 to 0.06). The same was observed in the continuous factorial MR and observational analyses. In conclusion, this study did not find evidence of interaction between BMI and 25(OH)D on the risk of psoriasis. Given minor differences in measured BMI and 25(OH)D between the factorial groups, small effects may have been undetected.

## Introduction

Psoriasis is a chronic, inflammatory skin disease that is associated with multiple comorbidities and has considerable impact on quality of life (Armstrong and Read, 2020). The prevalence varies between populations and geographical locations and is estimated to be 2–4% among adults in Western countries (Parisi et al, 2020). However, life-time prevalence as high as 6.6–11.4% has been reported in Norway (Danielsen et al, 2013; Solvin et al, 2023). Some data indicate increasing prevalence over the past decades (Danielsen et al, 2013; Iskandar et al, 2021 Solberg, 2023).

The obesity epidemic may partly explain the rising psoriasis prevalence because studies have consistently demonstrated an association between obesity and increased risk of psoriasis (Armstrong et al, 2012; Paroutoglou et al, 2020; Snekvik et al, 2017). Previous Mendelian randomization (MR) studies, including European and Japanese populations, have demonstrated a causal relationship between higher body mass index (BMI) and an increased risk of psoriasis (Budu-Aggrey et al, 2019; Ogawa et al, 2019), where reverse causality was found to be unlikely (Budu-Aggrey et al, 2019).

Vitamin D has several effects of importance to skin physiology; of particular relevance to psoriasis is regulation of the immune system as well as proliferation and maturation of keratinocytes (Umar et al, 2018). These physiological effects are utilized when treating psoriasis with topical vitamin D analogs (Armstrong and Read, 2020). Observational studies have found an association between lower serum 25-hydroxyvitamin D (25[OH]D) and psoriasis (Hambly and Kirby, 2017). MR studies have additionally demonstrated a causal relationship between lower 25(OH)D and an increased risk of psoriasis (Zhang et al, 2022; Zhao et al, 2023). Moreover, no reverse causal link has been observed (Drodge et al, 2021).

Recent observational studies suggest that the association between 25(OH)D and psoriasis may be modified by adiposity (BMI and hip–waist ratio) (Jenssen et al, 2024; Kuang et al, 2020; Zhang et al, 2022). No previous studies have explored this possible combined effect (ie, interaction) using MR designs, which are considered more robust to residual confounding and reverse causality than traditional observational approaches (Smith and Hemani, 2014). In factorial MR, genetic variants are used as instrumental variables to assess interaction between modifiable risk factors (North et al, 2019; Rees et al, 2020). Understanding relative excess (disease) risk due to interaction (RERI) can have implications for targeted interventions and public health strategies (Rothman et al, 2021).

In this study, we investigated RERI (referred to as interaction in the remaining parts of this paper) between genetically predicted BMI and 25(OH)D on psoriasis using observational data from 2 independent population-based cohorts: UK Biobank (UKB) and The Trøndelag Health Study (HUNT). We also explored interaction between measured BMI and 25(OH)D on the odds of psoriasis using a traditional cross-sectional design in UKB.

## Results

### General characteristics

The UKB sample included 398,404 individuals (53.8% women) with mean (SD) age of 56.9 (7.9) years, BMI of 27.4 (4.8) kg/m^2^, and season-adjusted 25(OH)D of 54.8 (19.7) nmol/l (Table 1). The HUNT sample included 86,648 individuals (53.0% women) with mean (SD) age of 46.1 (16.9) years and BMI of 26.4 (4.4) kg/m^2^ (Table 1).

**Table 1:** Characteristics of the Participants, UK Biobank (n = 398,404^1^) and HUNT (n = 86,648)

Characteristic	UK Biobank		HUNT	
	No Psoriasis	Psoriasis^2^	No Psoriasis	Psoriasis^2^
Number of participants	386,197/96.9	12,207/3.1	78,854/91.0	7,794/9.0
Age, y	56.9 (7.9)	57.2 (7.8)	46.2 (17.1)	44.7 (13.8)
Sex				
Female	208,122/53.9	6,056/49.6	41,674/52.8	4,275/54.8
Male	178,075/46.1	6,151/50.4	37,180/47.2	3,519/45.2
25(OH)D (nmol/l)^3^	54.8 (19.7)	53.3 (19.7)	NA	NA
BMI (kg/m^2^)	27.4 (4.7)	28.3 (5.1)	26.4 (4.3)	27.0 (4.6)

Abbreviations: 25(OH)D, 25-hydroxyvitamin D; BMI, body mass index; HUNT, The Trøndelag Health Study; NA, not applicable.

Continuous variables are given as mean (SD), and categorical variables are presented as number/proportions.

1 Dataset restricted (omitted participants missing genetic data + unrelated).

2 Psoriasis: medical records and/or self-report.

3 Season adjusted (Autumn mean).

The prevalence of psoriasis was 3.1% (n = 12,207) in UKB and 9.0% (n = 7794) in HUNT (Table 1). Participants with psoriasis had on average higher BMI (in both cohorts) and lower 25(OH)D (assessed in UKB only) than participants without psoriasis.

**Figure 1:**
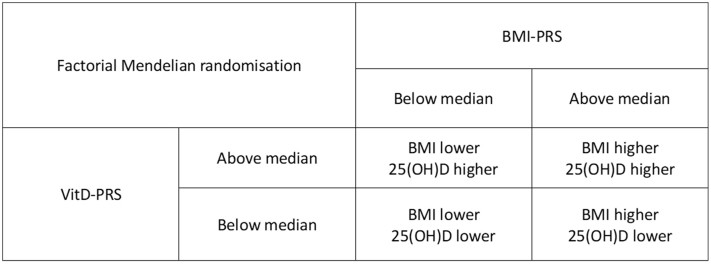
**Factorial Mendelian randomization using a 2 × 2 design.** This image was adapted from Rees et al (2020). BMI-PRS denotes the PRS for BMI, and vitD-PRS denotes the PRS for 25(OH)D. 25(OH)D, 25-hydroxyvitamin D; BMI, body mass index; PRS, polygenic risk score.

**Figure 2:**
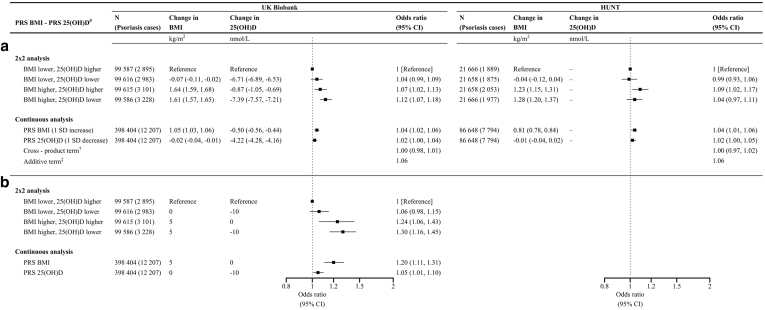
**OR estimates for psoriasis from factorial MR analyses in UKB and HUNT.** (**a**) Unscaled associations. (**b**) Associations in UKB scaled to represent a 5 kg/m^2^ increase in BMI and 10 nmol/l decrease in 25(OH)D. Upper panel (for **a** and **b**): Results from 2 × 2 factorial MR. Participants are separated in 4 groups on the basis of median PRS for BMI and 25(OH)D. RERI (95% CI) was estimated to −0.01 (−0.06 to 0.09) in UKB and −0.04 (−0.14 to 0.06) in HUNT. Meta-analysis of the UKB and HUNT result showed RERI (95% CI) of −0.015 (−0.046 to 0.048). Interaction exists if RERI ≠ 0. Lower panel (**a** and **b**): Results from continuous factorial MR, including the 2 PRSs on a continuous scale as well as the cross-product between PRSs. RERI (95% CI) was estimated to 0.00(-0.02, 0.02) in UKB and 0.00(-0.03, 0.02) in HUNT. Meta-analysis of the UKB and HUNT result showed RERI (95% CI) of 0.00 (−0.017 to 0.017). The symbols # denotes PRSs for BMI and 25(OH)D. In 2 × 2 analysis, PRSs were dichotomized at the median value and combined to 4 groups. OR estimates derived from logistic regression models adjusted for age, sex, genetic batch, and principal components 1–20. The symbol † denotes that continuous factorial MR included the PRS for BMI, PRS for 25(OH)D (both standardized to have a mean of 0 and SD of 1), and their cross-product. The symbol ‡ denotes the ORs for the additive term of the continuous variables calculated by exponentiating the sum of estimated beta values for PRS for BMI (b1), PRS for 25(OH)D (b2), and their cross-product (b3) (ie, OR = exp[b1+b2+b3]). N = total number of participants. 25(OH)D, 25-hydroxyvitamin D; BMI, body mass index; CI, confidence interval; HUNT, The Trøndelag Health Study; MR, Mendelian randomization; PRS, polygenic risk score; RERI, relative excess risk due to interaction; UKB, UK Biobank.

Table 2 displays general characteristics across the 2 × 2 factorial MR groups (Figure 1). Having a genetically predicted higher BMI and lower 25(OH)D combined corresponded to an average of 1.61 kg/m^2^ higher BMI and 7.39 nmol/l lower 25(OH)D in UKB and 1.28 kg/m^2^ higher BMI in HUNT (Figure 2). A 1 SD increase in the polygenic risk score (PRS) for BMI (referred to as BMI-PRS in the remaining parts of this paper) corresponded to a 1.05 and 0.81 kg/m^2^ increase in measured BMI in UKB and HUNT, respectively. A 1 SD decrease in the PRS for 25(OH)D (referred to as vitD-PRS in the remaining parts of this paper) corresponded to a 4.22 nmol/l lower measured 25(OH)D in UKB.

**Table 2:** Basic Characteristics of the Participants across the 2 × 2 Factorial Genetic Risk Groups, UKB (n = 398,404) and HUNT (n = 86,648)

Factorial Group	0	1	2	3
	BMI-PRS^1^ < Median vitD-PRS^2^ > Median	BMI-PRS^1^ < Median vitD-PRS^2^ < Median	BMI-PRS^1^ > Median vitD-PRS^2^ > Median	BMI-PRS^1^ > Median vitD-PRS^2^ < Median
**UKB**				
Number of participants	99,587	99,616	99,615	99,586
Psoriasis	2,895/2.9	2,983 / 3.0	3,101/3.1	3,228/3.2
Age	57.0 (7.9)	56.9 (7.9)	56.9 (7.9)	56.9 (7.9)
Sex				
Female	53,650/53.9	53,757/54.0	53,266/53.5	53,505/53.7
Male	45,937/46.1	46,859/46.0	46,349/46.5	46,081/46.3
BMI^3^	26.6 (4.5)	26.9 (4.5)	28.3 (5.0)	28.2 (5.0)
25(OH)D^4^	58.5 (20.9)	51.8 (17.8)	57.6 (20.9)	51.1 (17.8)
**HUNT**				
Number of participants	21,666	21,658	21,658	21,666
Psoriasis	1889/8.7	1875/8.7	2053/9.5	1977/9.1
Age	46.2 (17.0)	46.2 (17.0)	46.0 (16.8)	45.9 (16.8)
Sex				
Female	11,279/52.1	11,583/53.5	11,586/53.5	11,501/53.1
Male	10,387/47.9	10,075/46.5	10,072/46.5	10,165/46.9
BMI^5^	25.8 (4.0)	25.8 (4.1)	27.1 (4.5)	27.1 (4.6)

Abbreviations: 25(OH)D, 25-hydroxyvitamin D; BMI, body mass index; HUNT, The Trøndelag Health Study; PRS, polygenic risk score; UKB, UK Biobank.

Psoriasis was assessed from medical records and/or self-reported. 25(OH)D (nmol/L) was adjusted for season.

1 BMI-PRS, PRS for BMI: 940 SNPs in UKB/941 SNPs in HUNT.

2 VitD-PRS, PRS for 25(OH)D: 21 SNPs in UKB/19 SNPs in HUNT.

3 Missing value in 1262.

4 Missing value in 35,389.

5 Missing value in 638.

### Genetic instruments

The BMI-PRS (941 SNPs) explained 4.8% of the variation in BMI in UKB (R^2^ = 4.8%, F-statistic = 20,180) and 3.4% of the variation in BMI in HUNT (R^2^ = 3.4%, F-statistic = 3,065). It was weakly associated with 25(OH)D, age, and sex in the UKB and with age in HUNT (Table 3).

**Table 3:** Association between the PRS for BMI (BMI-PRS) and Possible Confounders of the Observational Association in UKB and HUNT

Possible Confounder	UKB		HUNT	
Dependent Variable^1^	β-Coefficient per 1-SD-Unit Increase in BMI-PRS^1^	95% CI	β-Coefficient per 1-SD-Unit Increase in BMI-PRS^1^	95% CI
25(OH)D	−0.03	−0.03 to −0.02	NA	NA
Age	−0.002	−0.004 to −0.001	−0.45	−0.76 to −0.13
Sex	0.0001	0.00002 to 0.0002	0.02	0.01 to 0.02

Abbreviations: 25(OH)D, 25-hydroxyvitamin D; BMI, body mass index; CI, confidence interval; HUNT, The Trøndelag Health Study; NA, not applicable; PRS, polygenic risk score; UKB, UK Biobank.

BMI-PRS denotes PRS for BMI.

1 β-coefficients from separate univariable linear models (ANOVA) for each of the listed dependent variables.

The vitD-PRS (21 SNPs) explained 4.6% of the variation in 25(OH)D in UKB (R^2^ = 4.6%, F-statistic = 17,490). It was weakly associated with BMI in the UKB but not in HUNT (Table 4).

**Table 4:** Association between the PRS for 25(OH)D (vitD-PRS) and Possible Confounders of the Observational Association in UKB and HUNT

Possible confounder	UKB		HUNT	
Dependent Variable^1^	β-Coefficient per 1-SD-Unit Increase in vitD-PRS^1^	95% CI	β-Coefficient per 1-SD-Unit Increase in vitD-PRS^1^	95% CI
BMI	0.02	0.006 to 0.03	−0.06	−0.19 to 0.07
Age	−0.001	−0.02 to 0.02	−0.007	−0.51 to 0.53
Sex	0.001	−0.0002 to 0.002	−0.01	−0.03 to 0.003

Abbreviations: 25(OH)D, 25-hydroxyvitamin D; BMI, body mass index; CI, confidence interval; HUNT, The Trøndelag Health Study; PRS, polygenic risk score; UKB, UK Biobank.

vitD-PRS denotes PRS for 25(OH)D.

1 β-coefficients from separate univariable linear models (ANOVA) for each of the listed dependent variables.

### Factorial MR analysis

In 2 × 2 factorial MR analyses, there were no evidence of relative excess risk for psoriasis due to interaction, neither in UKB (RERI = −0.01; 95% confidence interval [CI] = −0.06 to 0.09) nor in HUNT (RERI = −0.04; 95% CI = −0.14 to 0.06) (Figure 2 and Tables 5 and 6).

**Table 5:** A 2 × 2 Factorial Mendelian Randomization Analysis Comparing OR Estimates for Psoriasis Using Different Sets of PRSs for BMI and 25(OH)D, UK Biobank

BMI-PRS^1^	77			940		
vitD-PRS^1^	21	35	71	21	35	71
Group	OR (95% CI)	OR (95% CI)	OR (95% CI)	OR (95% CI)	OR (95% CI)	OR (95% CI)
Factorial group 0	1 (ref)	1 (ref)	1 (ref)	1 (ref)	1 (ref)	1 (ref)
Factorial group 1	1.05 (1.00–1.10)	1.04 (0.99–1.09)	1.05 (1.00–1.11)	1.04 (0.99–1.09)	1.07 (1.02–1.13)	1.03 (0.98–1.09)
Factorial group 2	1.03 (0.98–1.09)	1.02 (0.97–1.08)	1.05 (0.99–1.10)	1.07 (1.02–1.13)	1.10 (1.05–1.16)	1.08 (1.02–1.13)
Factorial group 3	1.07 (1.02–1.13)	1.08 (1.02–1.13)	1.06 (1.01–1.12)	1.12 (1.07–1.18)	1.13 (1.07–1.19)	1.11 (1.06–1.17)
	**RERI (95% CI)**	**RERI (95% CI)**	**RERI (95% CI)**	**RERI (95% CI)**	**RERI (95% CI)**	**RERI (95% CI)**
	−0.01 (−0.08 to 0.07)	0.02 (−0.06 to 0.09)	−0.03 (−0.11 to 0.04)	0.01 (−0.06 to 0.09)	−0.05 (−0.13 to 0.03)	0.01 (−0.07 to 0.08)

Abbreviations: 25(OH)D, 25-hydroxyvitamin D; BMI, body mass index; CI, confidence interval; PRS, polygenic risk score; ref, reference; RERI, relative excess risk due to interaction.

BMI-PRS denotes PRS for BMI, and vitD-PRS denotes PRS for 25(OH)D. All models adjusted for age, sex, genetic batch, and principal components 1–20. Interaction exists if RERI ≠ 0.

1 Digits refer to the number of SNPs included in the respective PRSs. For the BMI-PRSs, 77 of 77 and 940 of 941 selected SNPs were available, and for the vitD-PRSs, all selected variants were available. All PRSs were dichotomized at the median value and combined to 4 factorial groups, as follows: factorial group 0 = PRS-BMI below median, and PRS-vitD above median; expected to have the lowest risk for psoriasis and therefore chosen as ref group; factorial group 1 = PRS-BMI below median, and PRS-vitD below median; factorial group 2 = PRS-BMI above median, and PRS-vitD above median; and factorial group 3 = PRS-BMI above median, and PRS-vitD below median.

**Table 6:** A 2 × 2 Factorial Mendelian Randomization Analysis Comparing OR Estimates for Psoriasis Using Different Sets of PRSs for BMI and 25(OH)D, HUNT

BMI-PRS^1^	76			941		
vitD-PRS^1^	19	32	61	19	32	61
Group	OR (95% CI)	OR (95% CI)	OR (95% CI)	OR (95% CI)	OR (95% CI)	OR (95% CI)
Factorial group 0	1 (ref)	1 (ref)	1 (ref)	1 (ref)	1 (ref)	1 (ref)
Factorial group 1	0.96 (0.90–1.02)	1.02 (0.95–1.09)	0.98 (0.91–1.04)	0.99 (0.93–1.06)	1.00 (0.93–1.07)	0.95 (0.89–1.01)
Factorial group 2	1.07 (1.00–1.14)	1.09 (1.02–1.17)	1.06 (0.99–1.13)	1.09 (1.02–1.17)	1.06 (0.99–1.13)	1.02 (0.95–1.09)
Factorial group 3	1.06 (0.99–1.13)	1.10 (1.03–1.17)	1.08 (1.02–1.16)	1.04 (0.97–1.11)	1.08 (1.01–1.16)	1.07 (1.00–1.14)
	**RERI (95% CI)**	**RERI (95% CI)**	**RERI (95% CI)**	**RERI (95% CI)**	**RERI (95% CI)**	**RERI (95% CI)**
	0.03 (−0.07 to 0.12)	−0.01 (−0.11 to 0.09)	0.05 (−0.05 to 0.14)	−0.04 (−0.14 to 0.06)	0.03 (−0.07 to 0.12)	0.10 (0.01 to 0.19)

Abbreviations: 25(OH)D, 25-hydroxyvitamin D; BMI, body mass index; CI, confidence interval; HUNT, The Trøndelag Health Study; PRS, polygenic risk score; ref, reference; RERI, relative excess risk due to interaction.

BMI-PRS denotes PRS for BMI, and vitD-PRS denotes PRS for 25(OH)D. Interaction exists if RERI ≠ 0.

All models adjusted for age, sex, genetic batch and principal components 1–20.

1 Digits refers to the number of SNPs included in the respective PRS). For the BMI-PRSs, 76 of 77 and 941 of 941 selected SNPs were available, and for the vitD-PRSs, 19 of 21, 32 of 35, and 61 of 71 selected SNPs were available. All PRSs were dichotomized at the median value and combined to 4 factorial groups, as follows: factorial group 0 = PRS-BMI below median, and PRS-vitD above median; expected to have the lowest risk for psoriasis and therefore chosen as ref group; factorial group 1 = PRS-BMI below median, and PRS-vitD below median; factorial group 2 = PRS-BMI above median, and PRS-vitD above median; and factorial group 3 = PRS-BMI above median, and PRS-vitD below median.

Similarly, we observed no relative excess risk for psoriasis due to interaction in continuous factorial MR analysis assessing the combined effect of 1 SD increase in BMI-PRS (ie, genetic risk for higher BMI), 1 SD decrease in vitD-PRS (ie, genetic risk for lower 25[OH]D), and their cross-product neither in UKB (RERI = 0.00; 95% CI = −0.02 to 0.02) nor in HUNT (RERI = 0.00; 95% CI = −0.03 to 0.02) (Figure 2 and Tables 7 and 8).

**Table 7:** Continuous Factorial Mendelian Randomization Analyses Comparing OR Estimates for Psoriasis Using Different Sets of PRS for BMI and 25(OH)D, Included in the Model as Standardized Continuous Variables and their Cross-Product Term, UK Biobank

BMI-PRS^1^	77			940		
vitD-PRS^1^	21	35	71	21	35	71
Group	OR (95% CI)	OR (95% CI)	OR (95% CI)	OR (95% CI)	OR (95% CI)	OR (95% CI)
BMI-PRS (per 1 SD increase)	1.03 (1.01–1.05)	1.03 (1.01–1.05)	1.03 (1.01–1.05)	1.04 (1.02–1.06)	1.04 (1.02–1.06)	1.04 (1.02–1.06)
VitD-PRS (per 1 SD decrease)	1.02 (1.00–1.04)	1.02 (1.00–1.04)	1.02 (1.00–1.04)	1.02 (1.00–1.04)	1.02 (1.00–1.04)	1.02 (1.00–1.04)
Cross-product (BMI-PRS × vitD-PRS)	1.00 (0.99–1.02)	1.00 (0.98–1.02)	1.00 (0.98–1.02)	1.00 (0.98–1.01)	0.99 (0.97–1.01)	1.00 (0.98–1.02)
	**OR**	**OR**	**OR**	**OR**	**OR**	**OR**
Additive term (BMI-PRS + vitD-PRS + cross product^2^)	1.06	1.05	1.05	1.06	1.05	1.06
	**RERI (95% CI)**	**RERI (95% CI)**	**RERI (95% CI)**	**RERI (95% CI)**	**RERI (95% CI)**	**RERI (95% CI)**
	0.00 (−0.01 to 0.02)	0.00 (−0.02 to 0.02)	0.00 (−0.02 to 0.02)	0.00 (−0.02 to 0.02)	−0.01 (−0.03 to 0.01)	0.00 (−0.02 to 0.02)

Abbreviations: 25(OH)D, 25-hydroxyvitamin D; BMI, body mass index; CI, confidence interval; PRS, polygenic risk score; ref, reference; RERI, relative excess risk due to interaction.

All models adjusted for age, sex, genetic batch, and principal components 1–20. BMI-PRS denotes PRS for BMI, and vitD-PRS denotes PRS for 25(OH)D. Interaction exists if RERI ≠ 0.

1 Digits refers to the number of SNPs included in the respective PRSs. For the BMI-PRSs, 77 of 77 and 940 of 941 selected SNPs were available, and for the vitD-PRSs, all selected variants were available.

2 Calculated by exponentiating the sum of estimated β-values for BMI-PRS, vitD-PRS, and their cross-product (exp[b1+b2+b3]).

**Table 8:** Continuous Factorial Mendelian Randomisation Analyses Comparing OR Estimates for Psoriasis Using Different Sets of PRSs for BMI and 25(OH)D, Included in the Model as Standardized Continuous Variables and their Cross-Product Term, HUNT

BMI-PRS^1^	76			941		
vitD-PRS^1^	19	32	61	19	32	61
Group	OR (95% CI)	OR (95% CI)	OR (95% CI)	OR (95% CI)	OR (95% CI)	OR (95% CI)
BMI-PRS (per 1 SD increase)	1.05 (1.02–1.07)	1.05 (1.02–1.07)	1.05 (1.02–1.07)	1.04 (1.01–1.06)	1.04 (1.01–1.06)	1.04 (1.01–1.06)
vitD-PRS (per 1 SD decrease)	1.02 (1.00–1.05)	0.99 (0.97–1.02)	0.98 (0.96–1.00)	1.02 (1.00–1.05)	0.99 (0.97–1.02)	0.98 (0.96–1.00)
Cross-product (BMI-PRS × vitD-PRS)	1.00 (0.97–1.02)	1.00 (0.98–1.03)	1.00 (0.97–1.02)	1.00 (0.97–1.02)	1.00 (0.97–1.02)	1.00 (0.97–1.02)
	**OR**	**OR**	**OR**	**OR**	**OR**	**OR**
Additive term (BMI-PRS + vitD-PRS + cross product^2^)	1.07	1.04	1.02	1.06	1.03	1.01
	**RERI (95% CI)**	**RERI (95% CI)**	**RERI (95% CI)**	**RERI (95% CI)**	**RERI (95% CI)**	**RERI (95% CI)**
	−0.00 (−0.03 to 0.02)	0.00 (−0.02 to 0.03)	−0.00 (−0.03 to 0.02)	−0.00 (−0.03 to 0.02)	−0.01 (−0.03 to 0.02)	−0.00 (−0.03 to 0.02)

Abbreviations: 25(OH)D, 25-hydroxyvitamin D; BMI, body mass index; CI, confidence interval; HUNT, The Trøndelag Health Study; PRS, polygenic risk score; ref, reference; RERI, relative excess risk due to interaction.

BMI-PRS denotes PRS for BMI, and vitD-PRS denotes PRS for 25(OH)D. Interaction exists if RERI ≠ 0. All models adjusted for age, sex, genetic batch, and principal components 1–20.

1 Digits refers to the number of SNPs included in the respective PRS. For the BMI-PRSs, 76 of 77 and 941 of 941 selected SNPs were available, and for the vitD-PRSs, 19 of 21, 32 of 35, and 61 of 71 selected SNPs were available.

2 Calculated by exponentiating the sum of estimated β-values for BMI-PRS, vitD-PRS, and their cross-product (exp[b1+b2+b3]).

#### Sensitivity analysis

In sensitivity analysis, no substantial differences in the RERI estimates were apparent across PRS combinations in the 2 × 2 approach (Tables 5 and 6). In the continuous approach, RERI estimates were close to identical across PRS combinations (Tables 7 and 8).

RERI estimates are valid when all estimated ORs are above 1 (Knol et al, 2011). In the 2 × 2 MR analysis in HUNT, some of the combination has an estimated OR below 1 for the factorial group 1 in combinations including the focused (19 SNPs) and the extended (61 SNPs) vitD-PRS. These RERI estimates may not be valid and must be interpreted with caution. We therefore refrain from trusting the borderline significant RERI estimate for the 941-61 combination in HUNT (Table 6).

### Traditional observational analysis

In UKB, a 10 nmol/l decrease in measured serum 25(OH)D was associated with 3% higher odds for psoriasis (OR = 1.03; 95% CI = 1.02–1.04), and a 5 kg/m^2^ increase in measured BMI was associated with 19% higher odds for psoriasis (OR = 1.19; 95% CI = 1.17–1.22) (Table 9). The combined effect of the 2 factors summed to 22% higher odds for psoriasis (OR = 1.22; 95% CI = 1.09–1.37), supporting exact additivity in the observational association (RERI = 0.00; 95% CI = −0.01 to 0.03) (Table 9). A similar trend was found when scaling the factorial MR analysis (Figure 2).

**Table 9:** ORs for Psoriasis per 10 nmol/l Decrease in Measured Serum 25(OH)D and 5 kg/m^2^ Increase in Measured BMI, UKB

Predictor	OR (95% CI)^1^	OR (95% CI)^1^
25(OH)D, per 10 nmol/l decrease	1.03 (1.02–1.04)	1.03 (0.98–1.09)
BMI, per 5 kg/m^2^ increase	1.19 (1.17–1.22)	1.18 (1.13–1.25)
BMI × 25(OH)D (cross-product)		1.00 (0.99–1.01)
Additive interaction between 25(OH)D and BMI^2^		1.22 (1.09–1.37)

RERI (95% CI)
0.00 (−0.01 to 0.03)

Abbreviations: 25(OH)D, 25-hydroxyvitamin D; BMI, body mass index; CI, confidence interval; PRS, polygenic risk score; ref, reference; RERI, relative excess risk due to interaction.

Presented is a cross-sectional analysis of data from the UK Biobank (n = 12,207 psoriasis/386,197 nonpsoriasis^3^); 25(OH)D is in nmol/l, and BMI is in kg/m^2^. Interaction exists if RERI ≠ 0.

1 Logistic regression model adjusted for age and sex.

2 Calculated by exponentiating the sum of estimated beta-values for 25(OH)D, BMI and their cross-product (exp[b1+b2+b3]).

3 Counts differ from the Mendelian randomization analyses because of missing measured values (BMI, n = 1262; 25(OH)D, n = 35,247).

Support for exact additivity was also found in 2 × 2 observational analysis; having both 25(OH)D < 25 nmol/l and BMI > 27.5 was associated with 59% higher odds for psoriasis (OR = 1.59; 95% CI = 1.40–1.80) than having both 25(OH)D ≥ 25 nmol/l and BMI ≤ 27.5 (RERI = 0.05; 95% CI = −0.21 to 0.32) (Table 10). The single effect of having serum 25(OH)D < 25 nmol/l was associated with 21% higher odds for psoriasis (OR = 1.21; 95% CI = 1.09–1.33), and the single effect of BMI > 27.5 kg/m^2^ was associated with 38% higher odds for psoriasis (OR = 1.38; 95% CI = 1.33–1.43).

**Table 10:** ORs for Psoriasis in Groups Based on Dichotomized Measured BMI and 25(OH)D Levels, UKB

Associations	BMI > 27.5	25(OH)D < 25^1^	Psoriasis	No psoriasis	OR (95% CI) (Unadjusted)	OR (95% CI) (Adjusted^1^^,^^2^)
Single	—	No	10,669	339,125	1 (reference)	1 (reference)
	—	Yes	435	11,666	1.19 (1.07–1.31)	1.21 (1.09–1.33)
	No	—	5449	220,938	1 (reference)	1 (reference)
	Yes	—	5655	165,259	1.40 (1.35–1.46)	1.38 (1.33–1.43)
Combined	No	No	5287	196,501	1 (reference)	1 (reference)
	No	Yes	162	5,255	1.14 (0.98–1.34)	1.16 (0.99–1.35)
	Yes	No	5382	14,2624	1.40 (1.35–1.46)	1.38 (1.33–1.43)
	Yes	Yes	273	6,411	1.58 (1.40–1.79)	1.59 (1.40–1.80)
					**RERI (95% CI)**	**RERI (95% CI)**
					0.03 (−0.23 to 0.30)	0.05 (−0.21 to 0.32)

Abbreviations: 25(OH)D, 25-hydroxyvitamin D; BMI, body mass index; CI, confidence interval; RERI, relative excess risk due to interaction.

Presented is a cross-sectional analysis of observational data from UK Biobank (n = 12,207 psoriasis/386,197 nonpsoriasis^3^). BMI is in kg/m^2^, and 25(OH)D is in nmol/l. Interaction exists if RERI ≠ 0.

1 Season-adjusted 25(OH)D levels.

2 Adjusted for age and sex.

3 Counts differ from factorial Mendelian randomization analyses because of missing measured values (BMI, n = 1262; 25[OH]D, n = 35,247).

## Discussion

### Main findings

In this factorial MR study, we found no evidence of relative excess risk for psoriasis due to interaction between genetically predicted higher BMI and lower 25(OH)D, neither in UKB nor in HUNT. Moreover, there was no evidence of relative excess risk for psoriasis due to interaction between measured BMI and 25(OH)D in the observational data from UKB. To our knowledge, no previous MR studies have investigated the interaction between BMI and 25(OH)D.

Previous MR studies investigating each factor separately have demonstrated a causal relationship between higher BMI (Budu-Aggrey et al, 2019; Ogawa et al, 2019) and lower 25(OH)D (Zhang et al, 2022; Zhao et al, 2023) and increased risk for psoriasis. These findings imply that strategies to reduce overweight and vitamin D deficiency in the population may influence the incidence of psoriasis. Complementing these previous findings, our results find a similar direction of effect, suggesting that targeting both risk factors may have larger effect than a single intervention, but benefit cannot be expected to exceed the additive effect of the 2. Although it is difficult to approximate the effect of interventions from MR estimates (Burgess et al, 2012; Ference, 2018; Swanson et al, 2017), they are valuable for testing causal associations and supporting effect direction of long-term interventions (Burgess et al, 2012). Public health recommendations for nutrition, physical activity, and vitamin D supplementation are relevant examples of such life-long “interventions,” which would be extremely difficult to evaluate in trials (which might need follow-up for several decades). A North American randomized controlled trial showed a small preventive effect of vitamin D supplements for 5 years on autoimmune disease in general (Hahn et al, 2022). It was, however, underpowered to provide evidence for prevention of psoriasis specifically because there were only 38 confirmed psoriasis cases among the ∼25,000 participants (Hahn et al, 2022). Longitudinal studies suggest that weight loss after bariatric surgery can prevent psoriasis in subjects with overweight (Mahil et al, 2019). To our knowledge, no study has evaluated whether other weight-reduction strategies have similar preventive effect.

Our findings do not support the effect modification between BMI and 25(OH)D suggested in previous observational studies (Kuang et al, 2020; Zhang et al, 2022). However, direct comparison must be done cautiously because inconsistencies may be explained by methodological differences; MR studies estimate the effect of small differences in life-long exposures, which may be incomparable with the impact of larger exposure differences evaluated over limited time periods in traditional designs. Traditional observational designs can be affected by bias due to confounding, reverse causation, and measurement error, and violated or weak assumptions may bias MR estimates (discussed further below). That said, we found no substantial differences between the RERI estimates from MR and traditional analyses in our study, supporting the robustness of our findings. However, we do recognize that potential interaction effects may be short term or manifested only at thresholds or extremes, such as very low 25(OH)D levels or in higher BMI categories. Moreover, interactions may only be relevant for disease activity in established psoriasis, as suggested by our recent study (Jenssen et al, 2024), and not for incident psoriasis.

It has previously been argued that overweight and vitamin D deficiency may act synergistically in driving inflammation in psoriasis, considering the link between obesity-associated low-grade inflammation and psoriasis and the possible role of vitamin D deficiency in the proinflammatory state in obesity (Jensen and Skov, 2016; Jenssen et al, 2024; Paroutoglou et al, 2020; Todosenko et al, 2021). However, this hypothesis is not supported by the present findings.

### Strengths and limitations

The validity of MR effect estimates depends on 3 core assumptions: (i) relevance, the instrument is robustly associated with the risk factor (exposure); (ii) independence, there is no association between the instrument and confounding factors; and (iii) exclusion restriction, the instrument is only associated with the outcome through the exposure (Davey Smith and Hemani, 2014). We confirmed robust associations between the PRSs for both 25(OH)D and BMI in UKB and BMI in HUNT. Limited measurements prevented us from testing the association with 25(OH)D in HUNT. In a previous study, a PRS based on 3 SNPs explained 3.7% of the variation of 25(OH)D levels in a HUNT subsample (n = ∼6300) (Mai et al, 2019). Evaluating the associations between PRSs and known confounders of the observational association is often used to partly assess the second assumption (Davey Smith and Hemani, 2014). In our study, the BMI-PRS was weakly associated with 25(OH)D, age, and sex, and the vitD-PRS was weakly associated with BMI in UKB. However, the coefficients were small relative to the main effect on BMI and 25(OH)D, respectively. We can however not exclude associations with unknown or unmeasured confounders. Furthermore, we recognize that the association between BMI-PRS and 25(OH)D as well as the vitD-PRS and BMI may have unknown effects on the interaction analysis.

When pleiotropy is present, the third assumption is violated (Davey Smith and Hemani, 2014). Both instruments in our primary analyses have previously been used in 1-sample MR with psoriasis as outcome and found to be robust to pleiotropy (Budu-Aggrey et al, 2019; Emerging Risk Factors Collaboration/EPIC-CVD/Vitamin D Estimating dose-response relationships for vitamin D with coronary heart disease, stroke, and all-cause mortality: observational and Mendelian randomisation analyses, 2021). Instruments compiling variants in genes with well-known functional links to the exposure reduce the risk of pleiotropy. Ideally, we would have included a such focused PRS for BMI as well. However, this was not attempted because most BMI-associated variants have unknown functions (Loos and Yeo, 2022; Speakman et al, 2018).

Factorial MR has low power to detect true interaction, even in large samples (North et al, 2019; Rees et al, 2020), and this is further reduced by the multiple testing burden in our study. The difference in measured BMI (∼1.6 kg/m^2^) and 25(OH)D (∼7 nmol/l) between the groups in the 2 × 2 factorial MR analysis may be too small to detect interaction effects in our study. Comparing groups with genetically predicted larger differences than what is possible using common variants could provide more robust answers. Moreover, in simulation studies, an extension of 2-stage least squares approach has been shown to be more efficient for analyzing interactions (North et al, 2019). However, such methods are not yet developed for binary outcomes (North et al, 2019).

As discussed earlier, our findings reflect assumingly stable contribution of genetic risk over the life course. This assumption may not hold because the genetic contribution to BMI or 25(OH)D may differ with the state of the individual (eg, with concurrent disease), at different time points during development, or according to environmental exposures (such as UV availability). It is also plausible that the effect of overweight/vitamin D deficiency on psoriasis risk varies through life. Our data did not enable us to incorporate such potential time-varying effects. Life-course studies measuring BMI and 25(OH)D at several time points may increase our understanding. Research on multiple sclerosis indicates crucial time points for the effect of vitamin D on disease risk during childhood or adolescence (Sundström and Salzer, 2015). To our knowledge, no such study on psoriasis risk has been performed.

Cohort studies tend to recruit the healthiest in the population (Jacobsen et al, 2012; Langhammer et al, 2012) and are thereby at risk for selection bias. However, we find it reasonable to assume that the potential effects of participation on BMI, 25(OH)D, and genetic variants are independent of outcome status in our study. Any substantial impact on relative differences is therefore unlikely (Szklo and Nieto, 2019). The consistency of our findings in both cohorts is also reassuring. Using a case definition combining self-report and different sources of diagnosis data, we aimed to include as many psoriasis cases as possible. This case definition has not been validated. Thus, the extent of outcome misclassification is unknown. It is however assumable that any expected outcome misclassification is nondifferential (ie, independent of both BMI, 25[OH]D, and genetic variants). This may have biased the estimated ORs toward the null (Rothman et al, 2021).

The less clear trend between factorial groups in the 2 × 2 analysis in HUNT may be attributed to higher random error due to the smaller sample size. In addition, the high degree of relatedness in the HUNT sample may result in overprecision and family-level bias in our estimate. However, we expect this to be minimal. Some variation between UKB and HUNT may also be due to differences in case definitions, because 55% of the UKB sample lacked primary care data, and that self-report in UKB only captures doctor-diagnosed psoriasis. The study includes only White subjects of European ancestry and therefore may not be generalizable to other populations.

One strength of our study is that the use of genetic variants as proxies reduces the potential for confounding compared with traditional observational studies. Second, we utilize large-scale population-based cohorts compiling thousands of cases and controls, and therefore, any large interaction effect is likely to be detected. We have shown consistent RERI estimates across 2 independent cohorts and between PRS combinations. Our RERI estimates show similar results in both 2 × 2 and continuous MR approaches as well as in the observational analysis.

In conclusion, the results from this factorial MR study suggest that there is no relative excess risk for psoriasis due to interaction between BMI and 25(OH)D. That is, the combined effect did not exceed the additive effect of the 2 factors. Interventions to reduce overweight and vitamin D deficiency in the population may both influence psoriasis incidence, but the benefit of combined intervention cannot be expected to exceed the additive effect of the 2. Considering the small differences in actual BMI and 25(OH)D between the factorial groups, small interaction effects or other threshold effects may have been undetected.

## Materials and Methods

### UKB

UKB is a prospective cohort study including data from approximately 500,000 adult United Kingdom citizens (aged 40–69 years at recruitment) enrolled between March 2006 and July 2010 (Sudlow et al, 2015). Participants (5.5% of invitees) completed a touchscreen questionnaire, a formal interview, standardized clinical examinations, and biological sampling. Genotyping was performed, and data were linked with health records, including hospital records, death registry, and general practitioner data. The latter was available for 45% of participants when we accessed data in May 2023. Details regarding data collection have been described previously (UK Biobank 2007). UKB data field identifications used in this study are listed in Box 1.

**Box 1:** UKB Data Fields Used in this Study

UKB data field	Field ID
Ethnicity (White British)	21,000
Sex	31
Date of first L40 reported (psoriasis)	131,742
Source of report of L40	131,743
Age at assessment	21,003
Body mass index	21,001
Serum 25-hydroxyvitamin D	30,890
Month of attending assessment centre	55
Genotype measurement batch	22,000
Genetic principal components	22,009

Abbreviations: ID, identification; UKB, UK Biobank.

**Figure 3:**
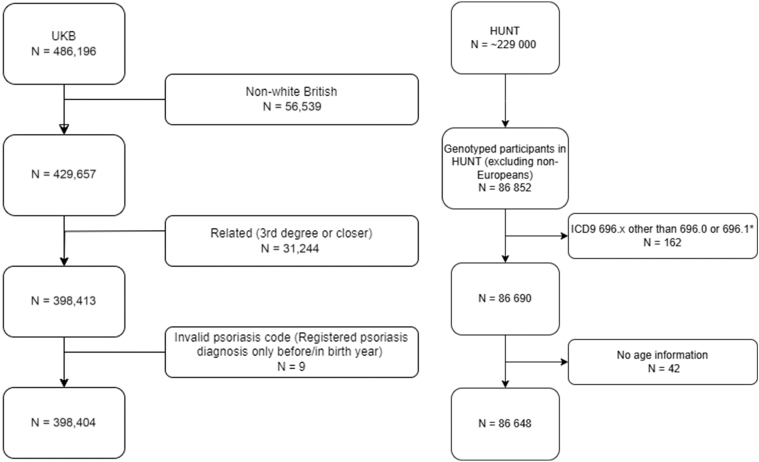
**Flow charts showing the study sample selection.** Left panel: UKB. Right panel: HUNT. The symbol ∗ denotes that participants with ICD9 codes 696.2 parapsoriasis, 696.3 pityriasis rosea, 696.4 pityriasis rubra pilaris, 696.5 other and unspecified pityriasis, or 696.8 other psoriasis and similar disorders were excluded. N = total number of participants. HUNT, The Trøndelag Health Study; ICD9, International Classification of Diseases, Ninth Revision; UKB, UK Biobank.

We restricted the UKB sample to include White British and unrelated individuals (kinship coefficient cut off = 0.0884) with complete data on the relevant SNPs and valid psoriasis code (flow chart in Figure 3). In total, 398,404 participants (214,178 women and 184,226 men) were included in the analysis.

### HUNT

HUNT is a population-based cohort study to which adults (aged ≥20 years at recruitment) in Trøndelag County, Norway are invited to repeated surveys (Åsvold et al, 2023; Krokstad et al, 2013). Data are collected through questionnaires, interviews, standardized clinical examinations, biological sampling, and linkage with health records. The number of participants (attendance proportion) was 77,202 (89.4%) in HUNT1 (1984–1986); 65,228 (69.5%) in HUNT2 (1995–1997); 50,800 (54.1%) in HUNT3 (2006–2008); and 56,042 (54.0%) in HUNT4 (2017–2019) (Åsvold et al, 2023). Among the participants in HUNT2–4, 88,615 individuals have been genotyped (Brumpton et al, 2022). We excluded non-European participants and those with invalid psoriasis code or missing information on age (flow chart in Figure 3). In total, 86,648 participants (45,949 women and 40,699 men) were included from the analysis.

### Classification of psoriasis

Participants with reported psoriasis either in medical records (International Classification of Diseases codes and/or codes from primary care) or by self-report were classified as having psoriasis.

In UKB, we used Date L40 first reported (psoriasis), which combines information from death registries, hospital records, general practitioner data, and self-report. The latter was registered if the participant mentioned psoriasis during the formal interview when asked about doctor-diagnosed serious illnesses or disabilities.

**Figure 4:**
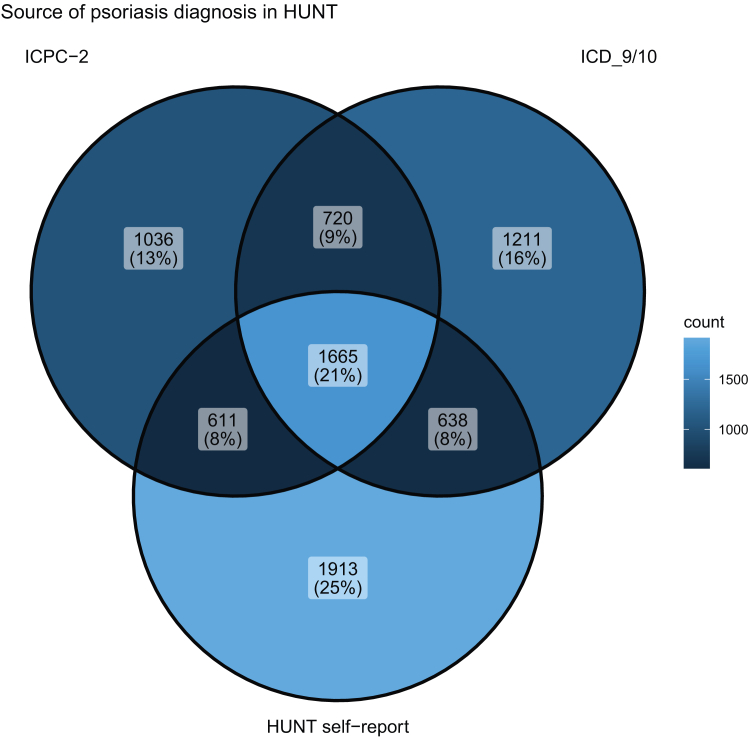
**Venn diagram showing the source of psoriasis diagnosis in HUNT.** Numbers are shown as count (%). HUNT, The Trøndelag Health Study; ICD10, International Classification of Diseases, Tenth Revision; ICD9, International Classification of Diseases, Ninth Revision; ICPC-2, International Classification of Primary Care, 2nd Edition.

In HUNT, International Classification of Diseases codes (International Classification of Diseases, Ninth Revision: 696.0/1; International Classification of Diseases, Tenth Revision: L40) were available from hospitals and private specialist practitioners and International Classification of Primary Care, second edition codes (S91) were available from general practitioners. Self-reported psoriasis was defined by answering “yes” to the questionnaire question “Have you had, or do you have psoriasis?” Self-reported psoriasis has acceptable validity in HUNT (positive predictive value = 78%; 95% CI = 69–85%) (Modalsli et al, 2016). The source of psoriasis diagnosis for the included participants are listed in Table 11 (UKB) and Figure 4 (HUNT).

**Table 11:** Source of Psoriasis Diagnosis UK Biobank

Source	n	%
Primary care only	4573	37.5
Primary care and other source(s)	863	7.1
Hospital admissions data only	1730	14.2
Hospital admissions data and other source(s)	64	0.5
Self-report only	2559	21.0
Self-report and other source(s)	2418	19.8

### Genetic instruments

In UKB, participants were genotyped using 1 of 2 genotyping arrays: Applied Biosystems UK BiLEVE Axiom Array or Applied Biosystems UK Biobank Axiom Array. In HUNT, genotyping was performed using 1 of 4 Illumina HumanCoreExome arrays: HumanCoreExome12, version 1.0; HumanCoreExome12, version 1.1; UM HUNT Biobank, version 1.0; or UM HUNT Biobank, version 2.0. The quality control and the imputation procedures have been described in detail previously (Brumpton et al, 2022; Bycroft et al, 2018; Mitchell et al, 2017).

PRSs summarize genetic information from several variants into 1 single variable, with the advantage of explaining a larger amount of variation in a trait than of single variants (Burgess and Thompson, 2013). We calculated PRSs for BMI and 25(OH)D (referred to as BMI-PRS and vitD-PRS, respectively, in this paper) for each participant as the sum of trait-increasing alleles, weighted by the GWAS effect size of the respective SNP (Burgess and Thompson, 2013). For our primary analysis, we developed a BMI-PRS combining 941 SNPs robustly associated with BMI in participants of European decent in the most recent GWAS meta-analysis (also including UKB participants) (Yengo et al, 2018). Of these, 940 variants were available in UKB, and all were available in HUNT. In sensitivity analysis, we developed another BMI-PRS combining 77 SNPs robustly associated with BMI in the most recent GWAS, which did not include UKB participants (Locke et al, 2015), and thereby avoiding internal weights (Burgess and Thompson, 2013). Of these, all were available in UKB, and 76 were available in HUNT.

For the vitD-PRS, we presented combinations of SNPs used in previous MR studies, where researchers have used different approaches in the selection of SNPs. In our primary analysis, the vitD-PRS included 21 SNPs from genes with well-known functions in the vitamin D metabolism (*CYP2R1*, *DHCD7*, *CYP24A1*, and *GC*), selected in a previous study by Estimating dose-response relationships for vitamin D with coronary heart disease, stroke, and all-cause mortality: observational and Mendelian randomisation analyses, 2021 using a step-wise procedure based on genome-wide significant association (*P* < 5 × 10^−8^) with 25(OH)D (Estimating dose-response relationships for vitamin D with coronary heart disease, stroke, and all-cause mortality: observational and Mendelian randomisation analyses, 2021). By selecting variants in genes with well-known functions, risk of pleiotropy is minimized (Estimating dose-response relationships for vitamin D with coronary heart disease, stroke, and all-cause mortality: observational and Mendelian randomisation analyses, 2021). In sensitivity analyses, we used a vitD-PRS, including 35 SNPs, selected in previous MR studies by Zhou and Hyppönen (2023) and Zhou et al (2022). They evaluated genome-wide significant SNPs associated with 25(OH)D in a recent GWAS (Revez et al, 2020) and selected those in which the association were replicated in an earlier GWAS (Jiang et al, 2018). Thus, they ensured the robustness in the GWAS signal in an external data source and avoided the use of internal weights (Burgess and Thompson, 2013; Zhou and Hyppönen, 2023). We also explored the impact of using a more extensive vitD-PRS, including 71 genome-wide significant SNPs from a recent GWAS (Manousaki et al, 2020), which were explored in the study by Emerging Risk Factors Collaboration/EPIC-CVD/Vitamin D Studies Collaboration Estimating dose-response relationships for vitamin D with coronary heart disease, stroke, and all-cause mortality: observational and Mendelian randomisation analyses, 2021. All selected variants were available in UKB. In HUNT, 19 of 21, 32 of 35, and 61 of 71 variants were available.

The SNPs and weights used in our analyses can be found in the Supplementary Excel File.

Both BMI-PRSs and the vitD-PRS used in our primary analyses have previously been used in 1-sample MR studies with psoriasis as outcome and found to be robust to pleiotropy (Budu-Aggrey et al, 2019; Emerging Risk Factors Collaboration/EPIC-CVD/Vitamin D Estimating dose-response relationships for vitamin D with coronary heart disease, stroke, and all-cause mortality: observational and Mendelian randomisation analyses, 2021).

### Other measurements

Age was defined by age at first inclusion. BMI was calculated as weight in kilograms per height in meters squared. Standing height and weight were measured at the assessment centers. For participants with repeated measurements, the first measurement was used. BMI was dichotomized using World Health Organization’s proposed threshold for public health action (BMI > 27.5 kg/m^2^) (WHO Expert Consultation, 2004).

Serum 25(OH)D was measured in UKB using a certified chemiluminescence immunoassay (DiaSorin LIAISON XL) (Lin et al, 2021). Nonfasting blood samples collected at the assessment centers were stored at −80 ^o^C before analysis. Season of blood draw was used for seasonal adjustment (December to February = Winter; March to May = Spring; June to August = Summer; September to November = Autumn). All measurements were converted to autumn measurement by subtracting the respective season mean and adding the autumn mean (eg, a summer value of 80 nmol/l was converted to season-adjusted value by this formula [80 − summer mean + autumn mean]) (Emerging Risk Factors Collaboration/EPIC-CVD/Vitamin D Estimating dose-response relationships for vitamin D with coronary heart disease, stroke, and all-cause mortality: observational and Mendelian randomisation analyses, 2021; Zhao et al, 2023). In HUNT, 25(OH)D measurements were only available for a limited number of participants (Sun et al, 2017) and therefore not included in the analysis.

### Statistical analyses

Logistic regression was used to estimate ORs for psoriasis using individual-level data separately in UKB and HUNT.

We dichotomized the BMI-PRS and vitD-PRS by the respective median value (separately for UKB and HUNT). Values equal to or below the median represented low PRS, and values above the median represented high PRS. Thereafter, participants were separated in 4 groups on the basis of the dichotomized BMI-PRS and vitD-PRS categories (Figure 1). We expected those with low BMI-PRS and high vitD-PRS (representing lower BMI and higher 25[OH]D) to have lowest odds for psoriasis and chose this group as reference.

We performed 2 × 2 factorial MR analyses using the 4 groups on the basis of the dichotomized BMI-PRS and vitD-PRS as a predictor variable. We also performed continuous factorial MR, including BMI-PRS, vitD-PRS (both standardized to have a mean of 0 and SD of 1), and their cross-product as predictors. All models included age, sex, genetic batch, and 20 principal components (Brumpton et al, 2022; Mitchell et al, 2017) (to account for population stratification). To ease comparison with measured BMI and 25(OH)D, we also present the OR estimates (UKB only) scaled to represent 5 kg/m^2^ increase in BMI and 10 nmol/l decrease in 25(OH)D. In response to peer review, we also performed meta-analyses of the RERI estimates from UKB and HUNT.

In UKB, we also performed cross-sectional analyses using observational data. In the continuous approach, predictors measured were 25(OH)D and BMI and their cross-product. In the 2 × 2 approach, the predictor was 4 groups based on dichotomized 25(OH)D (≥ or < 25 nmol/l) and BMI (> or ≤ 27.5 kg/m^2^) using 25(OH)D ≥ 25 nmol/l + BMI ≤ 27.5 kg/m^2^ as a reference group. Adjusted models included age and sex.

Interaction on an additive scale was assessed by estimating RERI (Knol et al, 2007; Rothman et al, 2021). An RERI estimate of zero supports exact additivity, that above zero supports positive interaction, and that below zero supports negative interaction.

Univariable linear models were applied to assess the association between the respective PRSs and measured 25(OH)D (UKB only) and BMI (UKB and HUNT). We evaluated F-statistics and R^2^ estimates to assess the strength of the genetic instruments. Moreover, we explored the association between the PRSs and possible confounders (BMI, age, sex) to assess the second MR assumption (ie, the instrument is not associated with confounders). In addition, the factorial MR was repeated with multiple PRSs to explore potential pleiotropy.

PLINK 2.0 was used to generate PRSs in UKB (Chang et al, 2015). Generation of PRSs in HUNT and all statistical analyses were performed in R, version 4.2.1 (R Foundation for Statistical Computing, Vienna, Austria). Estimates are reported with 95% CI.

## Ethics Statement

All participants have provided written informed consent. The National Health Service National Research Ethics Service approved the UK Biobank (reference 11/NW/0382). The Regional Committee for Medical and Health Research Ethics Central approved The Trøndelag Health Study. This study was conducted using the UK Biobank Resource under application number 40135 and was approved by the Regional Committee for Medical and Health Research Ethics, Mid-Norway (2015/2003).

## Data Availability Statement

Data may be obtained from a third party and are not publicly available. The UK Biobank resource is available to bona fide researchers for health-related research in the public interest (https://www.ukbiobank.ac.uk/enable-your-research). Access to data from The Trøndelag Health Study can be given to researchers with a PhD associated with Norwegian research institutes upon application to The Trøndelag Health Study’s Data Access Committee, given approval by a Regional Committee for Medical and Health Research Ethics. Researchers outside of Norway are welcome to apply for the use of The Trøndelag Health Study data in cooperation with a Norwegian Principal Investigator (https://www.ntnu.edu/hunt/data).

## ORCIDs

Marita Jenssen: http://orcid.org/0000-0003-1622-8491

Nikhil Arora: http://orcid.org/0000-0001-5767-5410

Mari Løset: http://orcid.org/0000-0003-3736-6551

Bjørn Olav Åsvold: http://orcid.org/0000-0003-3837-2101

Laurent Thomas: http://orcid.org/0000-0003-0548-2486

Ole-Jørgen Bekkevold Vassmyr: http://orcid.org/0000-0003-0109-4486

Xiao-Mei Mai: http://orcid.org/0000-0002-0426-7496

Yi-Qian Sun: http://orcid.org/0000-0002-9634-9236

Anne-Sofie Furberg: http://orcid.org/0000-0002-6311-166X

Rolf Jorde: http://orcid.org/0000-0002-8803-6408

Tom Wilsgaard: http://orcid.org/0000-0002-2709-9472

Kjersti Danielsen: http://orcid.org/0000-0002-6862-4632

Ben Michael Brumpton: http://orcid.org/0000-0002-3058-1059

## Conflict of Interest

KD reports to have served as a consultant or lecturer or participated in sponsored events/meetings by Novartis, Abbvie, LEO Pharma, UCB Pharma, Almirall, Meda Pharma, Bristol Myers Squibb, Galderma, and Celgene. The remaining authors state no conflict of interest.

## Supplementary Materials and Methods

List of SNPs included in the polygenic risk scores is presented in Supplementary Excel File.
